# Stage-Specific Animate Attention Bias in Individuals with High and Low Autistic Traits: Behavioral and Eye-Tracking Evidence

**DOI:** 10.3390/bs16050738

**Published:** 2026-05-09

**Authors:** Xinyu Zhao, Yaning Ji, Lin Li

**Affiliations:** 1 Faculty of Psychology, Tianjin Normal University, Tianjin 300387, China; 2230130220@stu.tjnu.edu.cn (X.Z.); jiyaning@stu.tjnu.edu.cn (Y.J.); 2 Key Research Base of Humanities and Social Sciences of the Ministry of Education, Academy of Psychology and Behavior, Tianjin Normal University, Tianjin 300387, China

**Keywords:** autistic traits, animate attention bias, eye-tracking, dot-probe task

## Abstract

Animate attention bias reflects the visual system’s tendency to prioritize animate over inanimate stimuli. This bias is reduced in autism spectrum disorder (ASD), suggesting that similar patterns may also be observed in individuals with high autistic traits (AT). Although previous research has reported reduced animate attention bias during early attentional orienting in individuals with high AT, how this bias unfolds across processing stages remains unclear. Using a dot-probe task combined with eye-tracking, the present study examined this stage-related pattern in individuals with high and low AT. Response time results showed that the low AT group had a significant animate-probe advantage, whereas the high AT group showed no significant advantage, broadly replicating prior findings. In stage-wise analyses, the low AT group showed a significant animate-probe advantage at the late stage, whereas the high AT group showed no significant advantage at either stage. However, this group difference was not reflected in most fixation-based measures. This RT–fixation dissociation suggests that reduced animate attention bias in high AT should not be interpreted simply as reduced overt fixation allocation to animate stimuli, but may reflect differences in using animacy-related cue-location information to facilitate subsequent probe detection and response selection.

## 1. Introduction

Animate attention bias reflects the tendency to rapidly detect and preferentially recognize animate information during cognitive processing, based on multiple sensory cues (New et al., 2007; Saito et al., 2023). This bias is consistent with the animate monitoring hypothesis, which suggests that the human visual system automatically monitors animate entities. This tendency is thought to have evolutionary origins and does not depend on current task requirements (New et al., 2007). Research indicates that certain animate-related cues emerge early in life, as infants show a preference for biological motion (Bardi et al., 2014; Bidet-Ildei et al., 2014; Rakison & Poulin-Dubois, 2001; Simion et al., 2008). In typically developing adults, the bias persists, manifesting as faster detection and localization of animate entities compared with inanimate ones (Pratt et al., 2010). In addition, the presence of animate entities reduces the accuracy of detecting inanimate entities. This effect becomes stronger when the animate and inanimate entities are spatially closer to each other (Altman et al., 2016; He & Cheung, 2019; Wang et al., 2015). From a developmental perspective, preferential attention to animate information is regarded as a fundamental component of social information processing (Klein et al., 2009; Scholl & Gao, 2013). Some socially relevant animate-related cues, such as biological motion, can attract and maintain attention, thereby providing perceptual input for subsequent social information processing (Frith & Frith, 1999; Popp & Serra, 2016; Rosa Salva et al., 2015). At the same time, evidence for a generalized animate attention advantage is not entirely consistent. For example, Hagen and Laeng (2016) showed that animal advantages in change detection can be influenced by scene context and target location, and Hagen and Laeng (2017) found that animal advantages in rapid visual presentation may reflect perceptual or reporting advantages rather than a uniform attentional effect. These mixed findings suggest that animacy-related advantages are not uniform across paradigms. For example, animacy advantages may be more robust in tasks that involve incidental detection or change monitoring, where automatic attentional capture plays a larger role, but may be attenuated under conditions of high perceptual load or when task demands emphasize deliberate search (Calvillo & Jackson, 2014; Hagen & Laeng, 2016). More broadly, they may depend on the interaction between task demands, perceptual load, stimulus context, and the specific processing stage or outcome measure being assessed. Thus, animate attention bias should not be treated as a single, invariant effect, but as a task-sensitive pattern that may be expressed differently in detection, response selection, and overt fixation allocation.

Given the foundational role of animate attention bias in social information processing, this automatic attentional mechanism has been examined in clinical populations, particularly among individuals with autism spectrum disorder (ASD). ASD is a neurodevelopmental condition characterized primarily by impairments in social communication and social interaction, accompanied by restricted and repetitive patterns of behavior, interests, or activities (American Psychiatric Association, 2013). These social difficulties may partly relate to atypical basic attentional processes, including reduced attention to socially relevant or animacy-related information (Thye et al., 2018). Studies examining animate attention in ASD have yielded mixed findings. Some studies suggest that individuals with ASD show reduced animate attention bias relative to typically developing individuals. For example, Moore et al. (2012) reported that typically developing individuals showed animate attention bias in a dot-probe task, whereas individuals with ASD did not. Eye-tracking studies have likewise reported that individuals with ASD allocate less visual attention to socially relevant stimuli, such as faces or people in scenes, than typically developing individuals. Studies using broader animacy-related materials have also reported reduced attention to such stimuli in ASD (Frazier et al., 2017; Klin et al., 2002; Klin et al., 2009; Rice et al., 2012). However, other studies have not found a reduced animate attention bias in ASD (New et al., 2010; Vanmarcke et al., 2017). Vanmarcke et al. (2017) reported comparable orienting responses to biological motion in individuals with ASD and typically developing controls. These inconsistencies may partly reflect methodological differences across studies. For example, studies using biological motion as stimuli may engage different perceptual and social-cognitive processes than those using static images of animals or naturalistic social scenes, making direct comparisons across paradigms difficult. In particular, variation in perceptual load may contribute to discrepant findings (Calvillo & Jackson, 2014), and differences in participants’ cognitive characteristics may also play a role. Taken together, these mixed findings suggest that reduced animate attention may not be a uniform characteristic across all autistic populations or task contexts.

Whether such reduced animate attention extends beyond clinically diagnosed ASD to individuals with high autistic traits remains an open question. Autistic traits (AT) refer to heritable social, communicative, and behavioral characteristics associated with ASD that are continuously distributed across the general population (Baron-Cohen et al., 2001; Jiang et al., 2024; Robinson et al., 2011; Sucksmith et al., 2011). Autistic traits and ASD are thought to share a common genetic and neurobiological basis (Bralten et al., 2018). This suggests that individuals with high AT may show some cognitive characteristics related to those observed in ASD. At the same time, high AT should not be equated with ASD. Individuals with high AT do not necessarily meet diagnostic criteria for ASD, and ASD may not be adequately understood as merely the quantitative extreme of typical variation (Frazier et al., 2023). Moreover, trait-defined samples of individuals with high AT may not be directly comparable to clinically ascertained ASD samples, as these groups can diverge in broader phenotypic and social-behavioral profiles (Banker et al., 2025). Even so, relatively intact social behavior in individuals with high AT does not mean that their social cognition is fully preserved. Some studies suggest that these individuals rely on compensatory strategies to maintain apparently typical social functioning (Forby et al., 2023; Guan & Zhao, 2015; Livingston et al., 2019; Palmer et al., 2015). In addition, core cognitive functions, such as empathy and cognitive flexibility, may still be less efficient (English et al., 2017; Jameel et al., 2015; Shirama et al., 2022; Stewart et al., 2023). Taken together, even when overt social behavior appears relatively intact, individuals with high AT may still differ in lower-level processing of socially relevant or animacy-related information.

Importantly, attentional processing is not a unitary process but comprises distinct temporal stages (Cisler & Koster, 2010), and reductions in animate attention bias may manifest differently across these stages. Recently, Yang et al. (2024) investigated attentional bias toward animate stimuli and its association with autistic traits. They proposed that overall response time mainly reflects the final outcome of attentional processing and may not capture dynamic differences between early orienting and later maintenance stages (Corbetta & Shulman, 2002; Panichello & Buschman, 2021; Petersen & Posner, 2012). To address this limitation, they adopted a modified dot-probe task with systematically varied cue durations: 100 ms and 300 ms to reflect early orienting, and 500 ms and 1000 ms to reflect later maintenance. Their findings supported the animate monitoring hypothesis in individuals with low AT, who showed animate attention bias at both stages. In contrast, this pattern was attenuated in individuals with high AT: reduced animate attention bias was evident during the early stage, and AT scores were negatively associated with animate attention bias only at this stage, not at the later stage. These findings suggest that reduced animate attention bias in high-AT individuals may be more evident during early attentional orienting. Although Yang et al. (2024) provided important initial evidence for understanding the relationship between autistic traits and animate attention bias, research on this issue remains relatively limited. Therefore, the first aim of the current study was to replicate prior evidence for reduced animate attention bias in individuals with high AT and to further examine how this pattern unfolds across processing stages.

To this end, we adopted the same temporal framework, with cue durations of 100 ms and 300 ms indexing relatively early attentional processing, and 500 ms and 1000 ms indexing later-stage attentional processing. This segmentation is grounded in the temporal dynamics of attention, as prior work suggests that attentional bias in the dot-probe task may reflect multiple component processes, including orienting, engagement, disengagement, and shifting (Chapman et al., 2019; Koster et al., 2004; Koster et al., 2005). Given that attentional shifts can occur within less than 100 ms (Buschman & Miller, 2009; Müller & Rabbitt, 1989), bias scores at 100–300 ms are more likely to reflect attention relatively close to initial orienting, before substantial disengagement and reallocation have occurred (Koster et al., 2005). By contrast, at 500 ms and beyond, attention has a greater opportunity to disengage and shift away from its initial target, so bias scores at these longer durations are more likely to reflect later-stage processes, including sustained maintenance or strategic reallocation, rather than initial orienting. It should be noted that this early–late distinction is an operational classification based on cue duration, rather than evidence for sharply discrete attentional stages. Attention unfolds continuously over time, and the present temporal bins provide only a coarse approximation of this dynamic process. Therefore, the stage-wise analyses should be interpreted as testing whether the animate-probe advantage differs between shorter and longer cue durations, rather than as identifying discrete early and late attentional mechanisms. The present study also employed an inverted stimulus condition. Inversion impairs visual recognition, particularly for biologically relevant stimuli such as faces (Yin, 1969). However, whether inversion attenuates the preferential processing of animate stimuli remains less clear (Rousselet et al., 2003). This manipulation, therefore, provides a preliminary way to assess whether the observed bias may depend on animate properties disrupted by inversion, rather than being driven solely by low-level visual properties.

However, the use of discrete cue durations in the dot-probe task may provide only a limited characterization of how animate attention bias unfolds over time. To address this limitation, eye-tracking technology provides highly sensitive indices of visual attention. Specifically, different eye-tracking indices capture distinct aspects of attentional processing during stimulus presentation. The probability and latency of the first fixation are commonly used to index early attentional orienting and initial attentional capture (Armstrong & Olatunji, 2012; Nummenmaa et al., 2006). First fixation duration reflects the initial attentional maintenance, whereas total fixation duration reflects sustained attention allocation (Just & Carpenter, 1980; Mayer et al., 2023; Rayner, 1979, 1998). Evidence from ASD research suggests that eye-tracking can reveal temporal features of attention to socially relevant animate stimuli that may be difficult to detect with response-time measures alone. Del Bianco et al. (2021) used continuous eye-movement recording and showed that typically developing individuals frequently move their fixations back to faces during the later stage of attention, reflecting dynamic differences in attention to socially relevant animate cues. In contrast, individuals with ASD rarely move their fixations back to faces after an initial rapid orienting, suggesting an alteration in the temporal dynamics of attention to socially relevant animate cues. Similarly, Hedger and Chakrabarti (2021) reported that individuals with ASD showed a lower proportion of fixations toward socially salient animate stimuli compared to typically developing individuals, and this proportion further decreased over time. These findings suggest that fixation patterns may provide a useful complement to response time measures by offering more direct information about visual attention during stimulus presentation. Therefore, the second aim of the present study was to use eye-tracking to complement response time measures and to examine fixation-based aspects of animate attention bias across stimulus presentation.

Based on the animate monitoring hypothesis and previous findings, we expected the low AT group to show a significant animate attention bias in response time, whereas this bias would be reduced or absent in the high AT group. At the stage level, we further expected this group difference to be more evident during the early stage than during the later stage. For the eye-tracking measures, we examined whether this group difference would also be reflected in fixation-based indices. Specifically, if animate attention bias extends to overt gaze behavior, the low AT group would be expected to show a greater likelihood of initially orienting toward animate stimuli, faster detection of animate stimuli, longer initial maintenance on animate stimuli, and a greater proportion of sustained attention allocated to animate stimuli, with these patterns reduced or absent in the high AT group. At the same time, RT-based and fixation-based measures may not always converge in the dot-probe task (Waechter et al., 2014). Therefore, the present study used eye-tracking to determine whether the RT-based animate-probe advantage reflected overt fixation allocation during cue presentation. If fixation-based measures did not show corresponding group differences, the RT effect would need to be interpreted more cautiously as a behavioral effect that was not directly mirrored in overt gaze allocation.

## 2. Materials and Methods

### 2.1. Transparency and Openness

The present research was conducted in accordance with the principles of the Declaration of Helsinki (General Assembly of the World Medical Association, 2014) and was approved by the Ethics Committee at the Faculty of Psychology, Tianjin Normal University (protocol number: APB2025060307, for study entitled “Stage-specific animate attention bias in individuals with high and low autistic traits: Behavioral and eye-tracking evidence”).

The response time and eye movement data are publicly available via: http://osf.io/n9rp3 (accessed on 2 May 2026).

### 2.2. Participants

Sample size was determined a priori using G*Power 3.1.9.7 (Faul et al., 2009). Setting statistical power to 0.80, α = 0.05, and assuming a medium effect size (f = 0.25) based on Cohen (1988), the required total sample size was calculated to be 34. Setting power to 0.90 increased this to 46, and setting it to 0.95 increased it to 54. We therefore recruited 49 participants per group (total *N* = 98), substantially exceeding the requirement for a power of 0.95 (*N* = 54), to ensure adequate statistical power to detect potential differences between groups.

Participants were college students from Tianjin recruited and screened using the Autism-Spectrum Quotient (AQ; Baron-Cohen et al., 2001). The AQ is a 50-item self-report instrument assessing autistic traits across five domains: social skills, attention switching, attention to detail, communication, and imagination. Items were rated on a 4-point scale ranging from 1 (definitely disagree) to 4 (definitely agree). For scoring, definitely agree and slightly agree responses are coded as 1, and slightly disagree and definitely disagree responses are coded as 0, yielding a maximum possible score of 50. The direction of scoring was reversed for negatively worded items. Higher scores indicate higher AT. The Chinese version of the AQ demonstrates good internal consistency (α = 0.82) and test–retest reliability, and has been validated for use in the general population (L. Zhang et al., 2016).

Following the classification method of Kunihira et al. (2006), a total of 196 questionnaires were administered for screening. This approach was adopted to maximize group differences in an initial exploratory investigation of animacy-related attentional bias. Participants who scored in the top 25% of the AQ distribution (AQ ≥ 26) were assigned to the High Autistic Traits (HAT) group (*n* = 49). Those scoring in the bottom 25% (AQ ≤ 22) were assigned to the Low Autistic Traits (LAT) group (*n* = 49). All participants provided written informed consent prior to the experiment and received monetary compensation upon completion.

All participants had normal or corrected-to-normal vision and were right-handed. The HAT group (9 males, 40 females) ranged in age from 18 to 24 years (*M* = 19.88, *SD* = 1.36) and had a mean AQ score of 29.82 (*SD* = 2.51). The LAT group (9 males, 40 females) ranged in age from 18 to 23 years (*M* = 19.61, *SD* = 1.19) and had a mean AQ score of 15.88 (*SD* = 2.45). As expected, the HAT group scored significantly higher on the AQ than the LAT group, *t* (96) = 27.816, *p* < 0.001. No significant differences were found between groups in age or gender distribution (see Table 1).

### 2.3. Apparatus and Stimuli

An EyeLink 1000 Plus eye tracker (SR Research, Ottawa, ON, Canada) was used to record gaze location monocularly (right eye) while participants viewed the display binocularly, at a sampling rate of 1000 Hz. This system provides high spatial resolution of <0.01° root mean square (RMS). A head and chin rest was employed to minimize head movements. Stimuli were presented on a 24-inch LCD monitor with a resolution of 1920 × 1080 pixels and a refresh rate of 150 Hz. Participants viewed the stimuli from a distance of approximately 75 cm.

Following Yang et al. (2024), six sets of images were selected from the standardized database by Moreno-Martínez and Montoro (2012), which is well-suited for animate–inanimate contrasts due to its categorical structure and controlled visual properties. To further control for low-level visual features, each animate image was paired with an inanimate image sharing a broadly similar overall shape. The resulting pairs were as follows: cat–lamp, kangaroo–guitar, horse–table, pigeon–sock, rooster–ice skates, and duck–rocking chair. Additional paired-samples tests based on the norms provided by Moreno-Martínez and Montoro (2012) showed no significant differences between animate and inanimate images in age of acquisition, familiarity, typicality, visual complexity, name agreement, or lexical frequency (*ps* > 0.05). The SHINE Toolbox was used to standardize the brightness and contrast of all images. To further examine whether low-level visual differences between animate and inanimate stimuli may have contributed to the observed effects, we compared the two categories on mean saliency, peak saliency, mean luminance, RMS contrast, and edge density using computational image analysis (OpenCV; Bradski, 2000). Wilcoxon signed-rank tests revealed no significant differences on any of these measures (all *ps* > 0.15).

**Figure 1:**
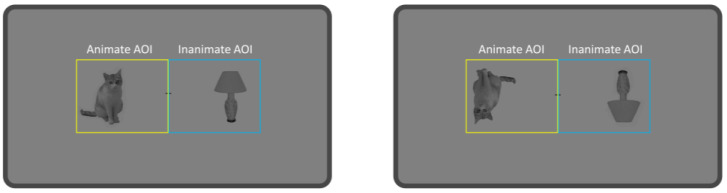
The upright and inverted materials of the experiment. The yellow and blue borders mark the left and right areas of interest (AOIs), respectively. Animate or inanimate images appeared randomly on the left or right side. The borders were not visible during the actual experiment.

All images were resized to 400 × 300 pixels (visual angle: 8.7° × 6.5°) and presented in pairs to the left and right of a central fixation point, with each image center positioned at a visual angle of 5.3° from the center of the screen. Images were presented on a gray background (RGB: 128, 128, 128) in both upright and inverted orientations (see Figure 1). Inverted stimuli were included as a preliminary manipulation to assess whether animate attention bias might depend on properties altered by inversion, rather than reflecting low-level visual properties alone.

### 2.4. Procedure

**Figure 2:**
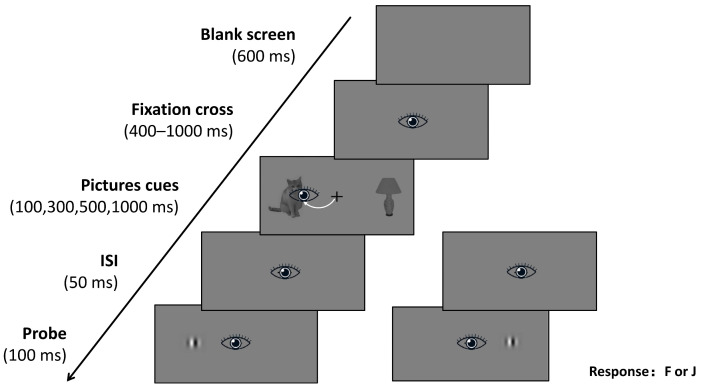
Experimental procedure. The eye icon indicates the fixation point location. The white dashed line indicates a possible eye movement path. The diagonal arrow indicates the temporal sequence of the experimental procedure.

A 2 × 2 × 4 × 2 mixed design was employed, with AQ group (HAT vs. LAT) as a between-subjects factor and probe type (animate-probe vs. inanimate-probe), cue duration (100, 300, 500, and 1000 ms), and cue orientation (upright vs. inverted) as within-subjects factors. For analysis purposes, the four cue durations were collapsed into two stages: early (100, 300 ms) and late (500, 1000 ms). Participants were tested individually. Prior to the experiment, they received written instructions describing the task procedure and completed eye-tracking calibration using a randomized 9-point method; the experiment commenced only after achieving a mean calibration error < 0.5°. The sequence of events in a single trial is illustrated in Figure 2. Each trial began with a central black fixation cross (visual angle: 0.8° × 0.8°) presented for a randomized duration of 400–1000 ms, during which participants were instructed to maintain fixation. Two images were then presented simultaneously to the left and right of the fixation cross, with each image centered at an eccentricity of 5.3°, for 100, 300, 500, or 1000 ms. After the images disappeared, a 50 ms interstimulus interval (ISI) followed. A probe dot (visual angle: 1.4° × 1.4°) was then displayed for 100 ms at the location previously occupied by either the left or right image. Participants were instructed to indicate the location of the probe as quickly and accurately as possible by pressing the ‘F’ key for left and the ‘J’ key for right. Each trial ended with a 600 ms blank screen. The complete session took approximately 30 min.

Before the main experiment, participants completed 32 practice trials. The main experiment comprised 192 trials. The experiment consisted of two blocks, one with upright images and the other with inverted images. Block order was counterbalanced across participants, such that half of the participants completed the upright block first and the other half completed the inverted block first. Within each block, trial order was randomized. Cue duration (100, 300, 500, or 1000 ms) varied randomly across trials, and the probe was equally likely to appear at the location of the animate or inanimate cue.

## 3. Results

### 3.1. Data Processing

#### 3.1.1. Response Time

All participants achieved high accuracy (>95%; HAT, *M* = 99.6%, *SD* = 0.06; LAT, *M* = 99.3%, *SD* = 0.08), with no significant difference between groups, *t* (96) = 1.71, *p* = 0.090, reducing concern about a speed–accuracy tradeoff. All participants were therefore included in the final analysis. For RT analysis, only correct trials were retained. Trials with response times shorter than 100 ms, longer than 1800 ms, or exceeding ±3 standard deviations from the mean were excluded. In total, 3.2% of trials were excluded.

#### 3.1.2. Eye Movement Data

To analyze eye-movement responses to the two images presented on each trial, two areas of interest (AOIs) were defined based on image orientation. For horizontally oriented image pairs (pigeon–sock and horse–table), each AOI spanned 390–690 px vertically; the left AOI spanned 340–990 px horizontally, and the right AOI spanned 990–1640 px horizontally. For vertically oriented pairs (cat–lamp, kangaroo–guitar, rooster–ice skates, and duck–rocking chair), each AOI spanned 340–740 px vertically; the left AOI spanned 440–990 px horizontally, and the right AOI spanned 990–1540 px horizontally. The interest period for eye-movement analyses was defined as the interval from stimulus onset to stimulus offset, so that all fixation metrics reflected attentional behavior during stimulus presentation only. Following standard procedures, fixations shorter than 80 ms or longer than 1200 ms were excluded (Rayner, 1998; M. Zhang et al., 2019).

Inspection of data quality across cue durations showed that, in the 100 ms condition, the rate of missing valid fixations was 100% in both groups, indicating that no interpretable fixation data could be obtained under this brief exposure duration. The 100 ms eye-tracking data were therefore excluded from further analyses. In the remaining conditions, missing rates were low: in the 300 ms condition, 0.94% for the HAT group and 0.72% for the LAT group (*p* = 0.634); in the 500 ms condition, 0.17% and 0.13% (*p* = 0.780); and in the 1000 ms condition, 0.13% and 0.04% (*p* = 0.312), respectively. No significant group differences in missing rates were observed in any condition, indicating comparable data quality between groups. After exclusion of the 100 ms condition, eye-movement data were classified into an early stage (300 ms) and a late stage (500 ms and 1000 ms), while retaining the early–late distinction used in the RT analysis.

To characterize different components of fixation allocation during stimulus presentation, four eye-movement bias indices were computed based on prior eye-tracking studies (Castellanos et al., 2009; Gao et al., 2011; Holas et al., 2014). The probability of the first fixation (PFF) was defined as the proportion of trials in which the first fixation landed on the animate image, indexing the initial direction of attention. The deviation of first fixation latency (DFFL) was calculated as the latency to first fixation on the animate image minus that on the inanimate image, with negative values indicating faster detection of animate stimuli. The deviation of first fixation duration (DFFD) was calculated as the first fixation duration on the animate image minus that on the inanimate image, with positive values indicating greater initial attentional maintenance for animate stimuli. The percentage of dwell time (PDT) was defined as the proportion of total fixation duration allocated to the animate image out of the total fixation duration allocated to both images, indexing sustained attentional allocation. PDT values above 0.5 indicated greater sustained attention to animate stimuli, whereas values below 0.5 indicated greater sustained attention to inanimate stimuli.

#### 3.1.3. Statistical Analyses

Data were analyzed using linear mixed-effects models (LMEMs; Baayen et al., 2008) with R and the lme4 package (version 4.3.2; Bates et al., 2011), and *p*-values were estimated using the lmerTest package (version 3.2.1; Kuznetsova et al., 2017). For binomial variables (e.g., accuracy), generalized LMEMs were conducted with the Laplace approximation. Contrasts were defined using sliding contrasts (the contr.sdif function) in the MASS package (Venables & Ripley, 2002). For RT analyses, AQ group, processing stage, cue orientation, and probe type served as fixed factors, with participants and items as crossed random effects. To examine potential effects of gender, additional analyses were conducted with gender included as a covariate in the LMEMs, entered as a fixed effect alongside the other predictors. For eye-movement bias score analyses, the AQ group, processing stage, and cue orientation served as fixed factors, with participant random intercepts included to account for repeated observations within individuals. Model diagnostics (Q–Q plots and residual-versus-fitted plots) were inspected for all retained models and indicated no substantial violations of normality or homoscedasticity. For the primary RT model, more complex random slopes were tested but produced singular fits and were therefore removed, retaining only random intercepts. For the eye-movement models, six stimulus pairs were insufficient for item-level random effects. The critical AQ group × probe type interaction remained significant when RTs were log-transformed. Effect sizes were calculated as Cohen’s d based on the residual standard deviation of the model, and 95% confidence intervals (CIs) were estimated using the Wald method. Statistical significance was determined based on the reported *p*-values. The analyses were organized according to an analytic hierarchy. The primary analysis was the RT-based LMEM testing the AQ group × probe type interaction, which examined whether the animate-probe advantage differed between the HAT and LAT groups. Stage-wise and orientation-specific RT analyses were conducted as follow-up analyses to characterize the temporal and orientation-related pattern of this effect. Eye-tracking analyses were treated as complementary exploratory analyses because they examined whether the RT-based group difference was accompanied by corresponding fixation-based differences during cue presentation. The AQ-continuous analysis was also exploratory and was used only as a robustness check within the restricted extreme-group sample, rather than as a full-dimensional analysis across the complete AQ distribution.

### 3.2. Response Time (RT) Analysis

Linear mixed-effects model analyses revealed a significant main effect of AQ group—*t* = −2.59, *p* = 0.011, 95% CI = [−74.996, −10.404], Cohen’s *d* = −0.553—with the HAT group showing longer overall RTs (*M* = 434 ms, *SD* = 134) than the LAT group (*M* = 392 ms, *SD* = 87). The main effect of the processing stage was also significant—*t* = 9.84, *p* < 0.001, 95% CI = [9.011, 13.493], Cohen’s *d* = 0.146—with longer RTs in the late stage (*M* = 419 ms, *SD* = 117) than in the early stage (*M* = 407 ms, *SD* = 112). Neither the main effect of cue orientation (*p* = 0.396) nor the main effect of probe type (*p* = 0.105) reached significance. Of the interaction terms, only the AQ group × probe type and AQ group × cue orientation interactions were significant; no other interactions reached significance (*ps* > 0.05).

The AQ group × probe type interaction was significant, *t* = 2.17, *p* = 0.030, 95% CI = [0.481, 9.445], Cohen’s *d* = 0.064. Simple effects analyses indicated that the LAT group responded significantly faster to animate-probe trials (*M* = 389 ms, *SD* = 86) than to inanimate-probe trials (*M* = 394 ms, *SD* = 87), *t* = −2.66, *p* = 0.008, 95% CI = [−7.481, −1.126], Cohen’s *d* = −0.056—consistent with an animate attention bias. In contrast, the HAT group showed no significant difference between probe types (animate-probe: *M* = 435 ms, *SD* = 134; inanimate-probe: *M* = 434 ms, *SD* = 133), *t* = 0.40, *p* = 0.692, 95% CI = [−2.534, 3.819], suggesting that no reliable animate attention bias was detected in this group. Additional analyses, including gender as a covariate, yielded the same overall pattern. The critical AQ group × probe type interaction remained significant, *t* = 2.06, *p* = 0.039, and gender did not interact with any experimental factor (*ps* > 0.15).

In addition, the AQ group × cue orientation interaction reached the conventional significance threshold, *t* = −1.98, *p* = 0.048, 95% CI = [−8.993, −0.036], Cohen’s *d* = −0.058. Simple effects analyses indicated that the LAT group showed significantly longer RTs in the inverted condition (*M* = 393 ms, *SD* = 88) than in the upright condition (*M* = 390 ms, *SD* = 85), *t* = 2.01, *p* = 0.045, 95% CI = [0.081, 6.435], Cohen’s *d* = 0.042, whereas the HAT group showed no significant difference between orientations (*p* = 0.420, 95% CI = [−4.480, 1.870]). To further characterize this interaction, the animate-probe advantage was examined separately within each group and orientation. In the LAT group, this contrast reached significance in the upright condition, *t* = 2.01, *p* = 0.045, 95% CI = [0.104, 9.102], Cohen’s d = 0.059, but not in the inverted condition, *t* = 1.75, *p* = 0.080, 95% CI = [−0.477, 8.502], Cohen’s d = 0.052. The HAT group showed no reliable animate-probe advantage in either orientation (upright: *t* = −0.45, *p* = 0.655; inverted: *t* = −0.12, *p* = 0.906).

As a follow-up analysis, we examined how the AQ group × probe type interaction unfolded across processing stages. Separate analyses were conducted for the early and late stages (see Table 2). In the early stage (100 ms and 300 ms), the LAT group showed faster responses to animate-probe (*M* = 385 ms, *SD* = 82) than inanimate-probe (*M* = 389 ms, *SD* = 84), but this difference did not reach significance, *t* = 1.82, *p* = 0.068. The HAT group showed no significant difference between probe types (*p* = 0.541). In the late stage (500 ms and 1000 ms), the LAT group responded significantly faster to animate-probe (*M* = 394 ms, *SD* = 89) than to inanimate-probe (*M* = 398 ms, *SD* = 90), *t* = 1.97, *p* = 0.049, indicating an animate attention bias. The HAT group again showed no significant difference between probe types (*p* = 0.945). Overall, stage-wise decomposition indicated that a statistically reliable animate-probe advantage was observed in the late-stage LAT analysis, whereas no reliable animate-probe advantage was detected in the HAT group at either stage.

To complement the extreme-group analysis, we conducted an exploratory AQ-continuous analysis within the experimental sample. The AT × probe type interaction was significant, *t* = −1.98, *p* = 0.048, 95% CI = [−0.605, −0.003], suggesting that higher AT scores were associated with a reduced RT-based animate-probe advantage. Given the extreme-group design, this finding should be interpreted as exploratory converging evidence rather than as a full-range dimensional analysis.

### 3.3. Eye Movement Measures

The following eye-tracking analyses were conducted as complementary exploratory analyses to examine whether the RT-based group difference was accompanied by corresponding fixation-based differences. Linear mixed-effects models were applied to each of the four eye-movement bias scores (PFF, DFFL, DFFD, and PDT). For each measure, an overall model with AQ group, processing stage, and cue orientation as fixed factors was first fitted, followed by stage-wise decomposition to further characterize whether any group differences varied across the early and late stages. A summary of the stage-wise group comparisons is presented in Table 3.

Probability of the First Fixation (PFF): In the full model, neither the main effect of AQ group nor any interaction reached significance (*ps* > 0.05). Decomposed by stage, the two groups did not differ in the early stage (HAT: *M* = 0.500, *SD* = 0.071; LAT: *M* = 0.500, *SD* = 0.069). In the late stage, the HAT group (*M* = 0.501, *SD* = 0.071) showed a significantly higher PFF than the LAT group (*M* = 0.481, *SD* = 0.068), *t* = −2.05, *p* = 0.042, Cohen’s *d* = −0.296 (see Table 3), indicating a group difference in the likelihood that the first fixation landed on the animate image.

Deviation of First Fixation Latency (DFFL): In the full model, no main effects or interactions reached significance (*ps* > 0.05). The mean DFFL was −0.160 (*SD* = 6.555) in the HAT group and 0.424 (*SD* = 5.909) in the LAT group. Stage-wise decomposition (see Table 3) revealed no significant group differences in either the early or late stage, indicating no reliable group difference in relative detection speed for animate versus inanimate stimuli.

Deviation of First Fixation Duration (DFFD): In the overall model, no main effects or interactions reached significance (*ps* > 0.05). The mean DFFD was −0.265 (*SD* = 50.303) in the HAT group and 0.940 (*SD* = 59.204) in the LAT group. Stage-wise decomposition (see Table 3) revealed no significant group differences in either the early or late stage, indicating no reliable group difference in initial attentional maintenance for animate relative to inanimate stimuli.

Percentage of Dwell Time (PDT): In the overall model, no main effects or interactions reached significance (*ps* > 0.05). The mean PDT was 0.501 (*SD* = 0.028) in the HAT group and 0.498 (*SD* = 0.030) in the LAT group, with both groups close to the no-bias level of 0.5. Stage-wise decomposition (see Table 3) revealed no significant group differences in either the early or late stage, indicating no reliable group difference in sustained attentional allocation to animate versus inanimate stimuli.

## 4. Discussion

The present study used a dot-probe task combined with eye-tracking to examine animate attention bias in individuals with high and low autistic traits across different cue durations. Response time analyses revealed that the LAT group responded significantly faster to probes replacing animate cues than to those replacing inanimate cues, reflecting an RT-based animate-probe advantage. In contrast, no such advantage was observed in the HAT group. Further stage-wise analysis of response times showed that the LAT group showed a significant animate-probe advantage only at the late stage, whereas the HAT group showed no reliable animate-probe advantage at either stage. However, this behavioral difference was not broadly mirrored by fixation-based measures during cue presentation. Given the small effect sizes of the RT contrasts and the largely null fixation-based findings, the observed group difference should be interpreted as a subtle RT-based effect rather than as evidence for a robust group difference in overt attentional allocation. Methodologically, this highlights the value of eye-tracking in clarifying whether RT-based attention-bias effects reflect overt fixation allocation.

The response time results are consistent with the animate monitoring hypothesis (New et al., 2007), which proposes that the human attention system has evolved to spontaneously monitor animate entities due to their adaptive significance. The LAT group responded faster to probes at animate cue locations, suggesting that animate stimuli were preferentially attended over inanimate stimuli. This advantage was absent in the HAT group, suggesting a reduced RT-based animate-probe advantage in individuals with high AT. These findings replicate the core pattern reported by Yang et al. (2024) and suggest that animate information may be processed differently in individuals with high and low AT. However, because the associated effect sizes were very small, this pattern should be interpreted as a modest RT-based difference rather than as a strong behavioral marker of autistic traits. Small effect sizes are characteristic of dot-probe measures of attentional bias; for example, a meta-analysis of 1005 clinically anxious individuals reported a mean dot-probe bias of only 1.8 ms (Cohen’s *d* = 0.05; Kruijt et al., 2019), partly reflecting the limited proportion of overall RT variance captured by the congruent–incongruent contrast.

Further analyses separating early and late processing stages revealed that only the LAT group exhibited a significant animate-probe advantage, and this was confined to the late stage. No reliable bias was detected in the HAT group at either stage. This pattern suggests that group differences in animate attention bias may not be limited to initial orienting, as later processing stages provide greater opportunity for attentional disengagement and reallocation (Koster et al., 2004). It is also compatible with findings from ASD research, suggesting that some group differences in attention to socially relevant animate stimuli may become more apparent during sustained viewing than during initial orienting (Del Bianco et al., 2021; Hedger & Chakrabarti, 2021). The present findings thus partially align with those of Yang et al. (2024), but show a different temporal pattern. Whereas Yang et al. (2024) emphasized an early-stage reduction in individuals with high AT, the present results suggest that the temporal expression of animate attention bias may not be fully captured by a strictly early-stage account (Cisler & Koster, 2010). However, this interpretation should remain cautious.

The present study included an inverted stimulus condition to provide a preliminary test of whether the animate-probe advantage depends on properties disrupted by inversion. Response time analyses revealed a significant interaction between the AQ group and stimulus orientation. The LAT group showed a significant animate-probe advantage in the upright condition, but this advantage was no longer significant in the inverted condition. The HAT group showed no detection advantage in either condition. This pattern suggests that the RT-based animate-probe advantage in the LAT group may partly depend on orientation-sensitive perceptual or configural information. However, the present pattern is informative in one respect: if the animate-probe advantage were driven primarily by low-level visual features, it should have persisted under inversion, because such features are largely preserved when images are rotated. The attenuation of this advantage in the inverted condition, therefore, suggests that the effect depends at least partly on orientation-sensitive configural properties. At the same time, because inversion disrupts configural processing while largely preserving semantic recognition, the present data cannot determine whether semantic-level categorization also contributes to the effect. Future studies could use scrambled stimuli, which disrupt both configural and semantic information, to further adjudicate between these accounts.

Although response time analyses revealed a significant group difference in animate attention bias, this pattern was not mirrored by most fixation-based measures. The deviation of first fixation latency, the deviation of first fixation duration, and the percentage of dwell time did not differ significantly between the HAT and LAT groups at either the early or late stage. This indicates that the present fixation-based measures did not detect reliable group differences in relative detection speed, initial attentional maintenance, or sustained attentional allocation. In the exploratory eye-tracking analyses, the one exception was the probability of first fixation at the late stage, where the LAT group directed fewer initial fixations toward animate images than the HAT group. This result ran counter to the overall expected pattern. However, PFF reflects only where the first fixation landed and does not necessarily indicate a sustained attentional preference. One possibility is that this isolated PFF effect reflects variability in first-fixation landing patterns during longer cue presentations, rather than a stable attentional preference for animate stimuli. It is also worth noting that the direction of this effect was opposite to the pattern expected under a reduced-orienting account. Specifically, the HAT group showed a higher PFF than the LAT group. This suggests that the finding does not reflect the same process underlying the RT-based group difference. This interpretation is supported by the absence of corresponding group differences in DFFL, DFFD, and PDT at the late stage, indicating that the PFF finding was not accompanied by reliable differences in relative detection speed, initial attentional maintenance, or sustained attentional allocation. Thus, this isolated PFF effect does not provide a coherent fixation-based explanation for the RT effect. Instead, it further shows how eye-tracking can constrain the interpretation of RT-based attention-bias effects when the two measures do not converge.

The dissociation between response time and eye-tracking measures observed in the present study is consistent with Waechter et al. (2014), who showed that RT-based and fixation-based bias indices in dot-probe tasks were largely uncorrelated. The present findings suggest that the reduced animate attention bias in high AT does not reflect reduced looking at animate stimuli, but rather a difference in how animacy-related spatial information is used to guide subsequent responses. The dot-probe task involves an initial presentation phase, during which participants allocate attention to the two images, and a subsequent response phase, during which participants detect and respond to the probe (Kappenman et al., 2014; Koster et al., 2004). Fixation-based indices mainly reflect overt fixation allocation during cue presentation, whereas RTs reflect the combined influence of cue processing, probe detection, and response selection. Thus, the RT-fixation dissociation provides a constraint on the interpretation of the RT effect. If the reduced animate-probe advantage in the HAT group reflected a broad reduction in overt attention to animate stimuli, corresponding group differences would be expected in fixation-based indices. However, this was not the case. The reduced RT-based animate-probe advantage may therefore reflect differences in how animacy-related cue-location information is used to facilitate subsequent probe detection and response selection, possibly through post-cue processes such as cue–probe mapping, rather than reduced overt fixation allocation to animate stimuli. This interpretation is also consistent with the possibility that fixation-based group differences may depend on task context. For example, passive viewing studies have reported reduced fixation time to animate stimuli in ASD relative to typically developing groups (Valiyamattam et al., 2020). Future studies could therefore compare dot-probe and passive viewing paradigms to determine whether animacy-related group differences emerge more clearly under spontaneous viewing than under speeded response demands.

These findings may also inform broader accounts of social cognition in relation to autistic traits. Previous research on high AT has largely focused on higher-order social-cognitive abilities such as theory of mind and empathy (Jameel et al., 2015; Stewart et al., 2023). The present results suggest that differences may also emerge at a more basic level. Both groups showed similar fixation patterns during cue presentation, yet differed in RT-based probe detection, indicating that the difference lies not in perceiving animacy-related information but in using it to guide subsequent responses. This pattern is consistent with a dimensional view of autistic traits, in which subtle processing differences are not confined to clinically diagnosed individuals but extend across the broader population. It also suggests that the scope of animacy-related processing differences in high AT may be wider than previously recognized, encompassing not only higher-order social inference but also more basic stimulus-response translation processes.

Beyond replicating the core RT pattern of Yang et al. (2024), the present findings help clarify what the reduced animate-probe advantage in high AT reflects. Previous RT-based studies have typically interpreted this reduction as indicating diminished attentional allocation to animate stimuli. However, the present eye-tracking results showed that the two groups did not differ in overt fixation allocation during cue presentation, suggesting that the RT-based group difference is unlikely to reflect a straightforward reduction in looking at animate stimuli. One possibility is that animate stimuli automatically attract covert spatial attention, strengthening the representation of their location even when overt fixation does not differ between animate and inanimate images. This enhanced spatial representation may facilitate the mapping between cue location and subsequent probe location, thereby speeding responses to probes appearing at animate-cue locations. In individuals with high AT, this covert facilitation may be less efficient: although they allocate similar overt fixation to animate stimuli, animacy-related spatial information may contribute less to response preparation. Under this interpretation, the group difference does not lie in how much attention is directed toward animate stimuli, as a perceptual-attentional account would suggest, but rather in how effectively animacy-related spatial information is used to guide responses. We refer to this as a processing-utilization account. In addition, the temporal locus of the group difference was not confined to the early stage, suggesting that the reduced animate-probe advantage cannot be fully captured by a strictly early-orienting account. More broadly, the present RT–fixation dissociation highlights that RT-based bias effects in the dot-probe paradigm should not be assumed to reflect overt attentional allocation without converging fixation-based evidence.

Several limitations constrain the interpretation of the present findings. First, the extreme-group design did not include experimental data from participants with mid-range AQ scores. Although the exploratory AQ-continuous analysis provided converging evidence within this sample, it cannot replace a full-dimensional test across the complete AQ distribution. Future studies should recruit participants across the full AQ range. Second, the use of only six stimulus pairs limits item-level generalizability. Although low-level visual properties were partially controlled through shape matching and luminance/contrast standardization, and psycholinguistic variables were checked using database norms, an additional analysis including stimulus pair and its interaction with probe type as fixed effects confirmed that the critical AQ group × probe type interaction remained significant (*t* = 2.07, *p* = 0.039), indicating that the effect was not driven by specific item pairs. Future studies should use larger and more diverse stimulus sets with more systematic control of perceptual and semantic properties. Third, the absence of valid fixation data in the 100 ms condition means that the eye-tracking early stage was based solely on the 300 ms condition, whereas the RT-based early stage included both 100 ms and 300 ms trials. This mismatch limits the comparability between RT and eye-tracking findings at the early stage, and the present study cannot determine whether RT-based effects at the shortest cue duration were accompanied by corresponding fixation-based differences. Accordingly, the RT–fixation dissociation observed here was established only for the 300 ms and longer conditions and may not generalize to the earliest stage of attentional orienting. Conclusions about whether the processing-utilization account applies to initial orienting remain open, and future studies should choose cue durations that ensure interpretable eye-tracking data across all time windows.

## 5. Conclusions

In conclusion, the present study showed that individuals with high autistic traits exhibited a reduced RT-based animate-probe advantage compared with those with low autistic traits, which replicated and extended the findings of Yang et al. (2024). The stage-wise RT pattern suggests that the group difference may not be fully captured by initial orienting alone. Importantly, the reduced RT-based animate-probe advantage was not clearly reflected in fixation-based measures, indicating that this effect should be interpreted as a subtle behavioral difference rather than as clear evidence for reduced overt attention to animate stimuli. These findings highlight the value of combining RT and eye-tracking measures within the dot-probe paradigm to clarify whether RT-based attention-bias effects are accompanied by corresponding differences in overt fixation allocation.

## Figures and Tables

**Table 1:** Participant characteristics by group (HAT vs. LAT).

Characteristics	HAT	LAT	Group Differences
Female (male)	40 (9)	40 (9)	χ^2^ (1) = 0.00, *p* = 1.000
Age range (years)	18–24	18–23	
Age	19.88 (1.36)	19.61 (1.19)	*t* (96) = 1.03, *p* = 0.307
AQ	29.82 (2.51)	15.88 (2.45)	*t* (96) = 27.82, *p* < 0.001

Note. HAT = High autistic traits, LAT = low autistic traits.

**Table 2:** Stage-wise probe-type comparisons of reaction time in the HAT and LAT groups.

Group & Stage	Animate *M* (*SD*)	Inanimate *M* (*SD*)	*b*	*t*/*z*	*p*	95% CI	Cohen’s *d*
HAT							
Early stage	428 (131)	427 (133)	−1.40	−0.61	0.541	[−5.88, 3.08]	−0.018
Late stage	441 (137)	441 (133)	0.16	0.07	0.945	[−4.32, 4.64]	0.002
LAT							
Early stage	385 (82)	389 (84)	4.17	1.82	0.068	[−0.31, 8.65]	0.054
Late stage	394 (89)	398 (90)	4.50	1.97	0.049 *	[0.02, 8.98]	0.058

Note. Response times are in milliseconds. Animate and inanimate refer to the probe type. *b* reports the model-based estimate for the probe-type contrast. Positive *b* values indicate faster responses to animate-probe trials relative to inanimate-probe trials. * *p* < 0.05.

**Table 3:** Stage-wise AQ-group comparisons for the eye-movement bias scores.

Contrast	Statistic	PFF	DFFL	DFFD	PDT
Early stage, HAT vs. LAT	*b*	0.02	0.07	1.44	0.01
	*SE*	0.01	1.53	15.03	0.01
	*t*/*z*	1.28	0.05	0.10	1.25
	*p*	0.203	0.966	0.932	0.338
	95% CI	[−0.01, 0.05]	[−2.92, 3.07]	[−28.01, 30.90]	[−0.01, 0.02]
	Cohen’s *d*	-	-	-	-
Late stage, HAT vs. LAT	*b*	−0.02	−0.52	5.71	−0.003
	*SE*	0.01	0.89	7.84	0.004
	*t*/*z*	−2.05	−0.59	0.73	−0.62
	*p*	0.042 *	0.555	0.467	0.539
	95% CI	[−0.04, −0.001]	[−2.27, 1.22]	[−9.67, 21.08]	[−0.01, 0.01]
	Cohen’s *d*	−0.296	-	-	-

Note. PFF = Probability of the first fixation; DFFL = deviation of first fixation latency; DFFD = deviation of first fixation duration; PDT = percentage of dwell time. A dash (-) indicates that Cohen’s *d* was not estimated for these comparisons. * *p* < 0.05.

## Data Availability

The data in this study are available from http://osf.io/n9rp3 (accessed on 2 May 2026).

## Abbreviations

The following abbreviations are used in this manuscript:

AT	Autistic traits
ASD	Autism spectrum disorder
AQ	Autism-spectrum quotient
RT	Response time
AOI	Area of interest
ISI	Inter-stimulus interval
HAT	High autistic traits
LAT	Low autistic traits
RMS	Root mean square
PFF	Probability of the first fixation
DFFL	Deviation of first fixation latency
DFFD	Deviation of first fixation duration
PDT	Percentage of dwell time
